# Acute and delayed effects of strength training in ball velocity and accuracy in young competition tennis players

**DOI:** 10.1371/journal.pone.0260825

**Published:** 2021-12-09

**Authors:** Manuel Terraza-Rebollo, Ernest Baiget

**Affiliations:** 1 Balearic Islands High Performance Sports Center (CTEIB), Palma de Mallorca, Balearic Islands, Spain; 2 National Institute of Physical Education of Catalonia (INEFC), University of Barcelona (UB), Barcelona, Spain; University of Mississippi, UNITED STATES

## Abstract

This study aimed to investigate the acute and delayed effects of medicine ball throws and resistance training in ball velocity and accuracy of serve, forehand and backhand in young competition tennis players. A crossover-randomized design was used with 10 competition tennis players (6 girls and 4 boys between 14 and 18 years old). The subjects performed 6 stroke test sessions, 3 for each strength protocol. The velocity and accuracy of strokes were measured before (basal situation), 3 minutes, 24 and 48 hours after the protocol. Medicine ball throws protocol was performed by accomplishing 3 sets of 6 repetitions using a 2 kg ball, throwing it at maximal speed. Resistance training protocol was performed by accomplishing 3 sets of 6 repetitions at 75% one-repetition maximum, lifting the load at maximal speed of bench press, dead lift, one hand row and half squat. There were no significant (p > 0.05) differences in all strokes, regarding ball velocity and accuracy after each method and each recovery time, compared to the basal situation. These results suggest that medicine ball throws and resistance training methods have no acute and delayed detrimental effects on stroke velocity and accuracy in young competition tennis players.

## Introduction

In tennis, like many sports, strength training is essential for a high tennis performance, not only to develop strength and power, but also to prevent injuries [1–3]. Due to the importance of ball velocity in the outcome match and the increase of it in the modern game [4, 5], strength should be trained in order to improve it [4, 6]. The ability to produce a high ball velocity is a key point for tennis players performance [7, 8], because serve has been positively correlated with the proportion of points won [9] and forehand and backhand groundstrokes ball velocity seems to be the determining factor that separates elite from sub-elite tennis players [10]. Besides ball velocity, accuracy is important in a successful play [2, 5], even a higher ball velocity and accuracy forehand has been associated with a higher experience of players [11]. Although it has been observed a negative correlation between serve velocity and the proportion of serve that are in [9], a high ball velocity and a high accuracy are required for the best performance, as a consequence, assessing both of them is important for stroke evaluation. In order to achieve an effective stroke involving a proper ball velocity and accuracy, a synchronised kinetic chain is essential [2, 3, 5], including ground reaction forces that are transferred through the feet, legs, trunk, upper body, and finally to the racket [3] using the stretch-shortening cycle in the main muscles [2]. Therefore, improving power is a key point in conditioning tennis training [3].

Several studies recommend medicine ball throws (MB) and resistance training (RT) methods to increase ball velocity in tennis [1–3, 6, 12] or overhead throw sports [13, 14]. Despite being two common methods in tennis strength training, which have shown improvements in tennis strokes when trained from 6 to 8 weeks [4, 5, 12] their acute and delayed effects in ball velocity and accuracy in competition tennis players had not been investigated until now. Related to the acute effects, it has not been observed post-activation potentiation effects in tennis serve velocity and accuracy performing complex training by using MB [15] or RT [16]. To our knowledge, no more studies investigated the acute effects of strength training in stroke tennis performance and no studies about acute and delayed effects were found.

Although the acute and delayed effects after a maximum and explosive strength training sessions have not been widely investigated in tennis, it seems that a maximum strength and rate of force development (RFD) loss in both isometric and dynamic contractions appears after explosive or maximal strength stimulus from 40% to 100% 1RM (one-repetition maximum) caused by central and peripheral fatigue [17–19]. Hence a ball velocity and accuracy impairment after a strength training session could be possible. These effects could be altered by several variables that establish the training stimulus such as exercise type and order, sets and repetitions number, rest duration, loading and velocity movement [20].

Therefore, the aim of this study was to investigate the acute and delayed effects of RT and MB in stroke ball velocity and accuracy in young competition tennis players. Our working hypothesis was that RT and MB could impair ball velocity, but would have no effect, or only a slight decrease, on accuracy, and that these impairments could be recovered after 24 or 48 hours. Also, RT may cause a greater decrease in ball velocity and accuracy due to the powerful nature of tennis strokes, the higher loads used and probably due to the higher time under tension.

## Materials and methods

### Participants

Ten competition tennis players (6 girls and 4 boys) between 14 and 18 years old and with at least 4 years of experience in competition tennis training participated in this investigation (Table 1). During the previous two months, all subjects practiced between 17.5 and 26 hours per week (24.3 ± 3.6 hours), performing between 10 and 17.5 hours (16.0 ± 3.1 hours) of specific tennis training (technical-tactical skills on-court) and between 7.5 and 8.5 hours (8.3 ± 0.4 hours) of fitness training.

**Table 1:** Participant characteristics.

	Whole group (n = 10)	Male (n = 4)	Female (n = 6)
Age (years)	15.3 ± 1.2	16.2 ± 1.1	14.7 ± 0.9
Body mass (kg)	57.6 ± 8.7	65.7 ± 8.1	52.2 ± 3.2
Height (cm)	168.1 ± 10.4	177.3 ± 9.6	162.0 ± 5.3
1 RM Bench press (kg)	40.6 ± 14.4	52.0 ± 15.6	33.0 ± 7.3
1RM Half squat (kg)	81.5 ± 25.1	101.3 ± 25.3	68.3 ± 15.2
1 RM Dead lift (kg)	73.4 ± 18.0	88.8 ±17.6	63.7 ± 10.6
1 RM Row dominant hand (kg)	31.1 ± 6.6	37.5 ± 2.6	26.8 ± 4.4
1 RM Row non dominant hand (kg)	29.0 ± 6.7	36.0 ± 2.7	24.3 ± 3.3

Values are mean ± standard deviation. 1 RM = one-repetition maximum.

All the subjects had over 4 years of experience in competition tennis training, did not practice another competitive sport, had not been injured in the last six months, knew the basics strength training exercises, and did not participate in another specific strength training program during the investigation. All participants volunteered to take part in the study and were previously informed of its aims, methods and potential risks. The subjects or their legal tutors for minors, signed an informed consent document prior to starting the study. This study was designed according to the Declaration of Helsinki of 1975, revised in 2008, and the Research Ethics Committee of the University of Vic–Central University of Catalonia approved the protocol (reference 21/2017).

This sample size was justified by a priori power analysis (using GPower Version 3.1.9.5, University of Dusseldorf, Dusseldorf, Germany) introducing the following parameters: effect size index (0.40) assuming a large partial eta-squared (0.14), α error probability (0.05), power (0.95), number of groups (1) and measurements (8), which resulted in a sample size of 10 subjects.

### Study design

**Fig 1:**
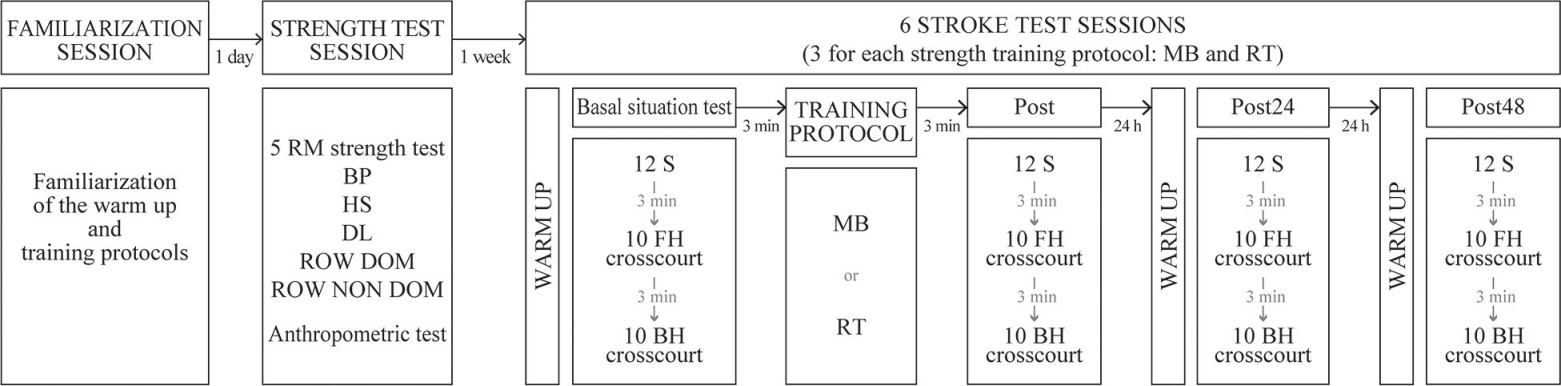
Study design chronology. MB = medicinal ball throws; RT = resistance training; BP = bench press; HS = half squat; DL = dead lift; ROW DOM = dominant hand row; ROW NON DOM = non-dominant hand row; S = serve; FH = forehand; BH = backhand.

A crossover-randomized design was used to investigate the acute (3 min) and delayed (24 and 48 h post-training) effects of two different strength protocols (MB and RT) on ball velocity and accuracy in young tennis players in off-season competition (from 8th January until 14th February). Subjects participated in 1 familiarization session, 1 strength test session (maximum strength test and anthropometric test) and 6 stroke test sessions performing 8 stroke tests (4 for each strength protocol: Basal situation test, Post, Post24 and Post48) (Fig 1). Each strength training protocol was separated by 7 days. On the first day of each strength protocol, participants completed one of both protocols in randomized order. Serve, forehand and backhand velocity and accuracy tests were measured before (basal situation), 3 min (Post), 24 (Post24) and 48 (Post48) hours after strength protocol. The tests were performed in two off-season microcycles.

### Procedures

One week prior to the intervention, the participants were asked to attend a familiarization session where they handed in the informed consent, the study protocol was explained, and they were instructed how to perform the exercises in proper form. The following day, the subjects completed the anthropometric test (body mass and height) and the 5-repetition maximum (5RM) strength test (strength test session), performing barbell bench press, barbell dead lift, dumbbell dominant one arm row and dumbbell non-dominant one arm row, and barbell half-squat (Table 1). Brzycki equation was used to calculate the 1RM [21]: 1RM = 100 * load rep / (102.78–2.78 * rep). Estimation strength test by 5RM was chosen in front of 1RM test because the subjects were familiar with it, there is a potentially lower risk of muscle injury and it has an easier participants preparation [22].

On the test day, the subjects began with a warm-up protocol that included 10 minutes of general warm-up activities (jogging, skipping, dynamic mobility and dynamic stretching) and 10 minutes of specific warm-up for tennis (exercises with elastic tubing for upper body, 10 dynamic serves, forehands and backhands imitations, five minutes baseline shots drill and 10 warm-up serves). Then, the basal situation test was performed. After 3 minutes, one of both strength training protocols was accomplished (MB or RT). Once finished, and after 3 minutes, the Post-Test was done to determine the acute effects (Post). The next day, they fulfilled the warm-up and 3 minutes later, the post 24 h test was performed (Post24). After two days, the same procedure was followed with the post 48 h test (Post48). Post24 and Post48 were done to determine the delayed effects (Fig 1). During the stroke test sessions, the subjects were allowed to drink water ad libitum. However, any supplementation was not allowed.

### Measurements

The stroke test consisted of assessing the peak ball velocity and accuracy of 12 flat serves (6 each side), 10 forehands and 10 backhands crosscourt (Fig 1), resting 20 seconds between serves, 10 seconds between forehands and backhands and 2 minutes between sets to avoid neuromuscular fatigue. The participants were constantly encouraged to hit the ball at maximum speed and high accuracy. No further information about the movement and no feedbacks about the performance were given during the set, but when the set was finished the subjects were informed about the velocity and accuracy achieved. Mean peak ball velocity and mean score accuracy were used for the analysis. Only the strokes that were “in” were registered for the velocity analysis. The tests were performed at the same time of the day for reliability reasons and to control the circadian variation. Moreover, the subjects were instructed to use the same racket, strings and string tension to perform the different tests. The subjects were required to refrain from any high intensity exercise on the day before each test day. All tests were performed on an outdoor clay tennis court.

A radar gun (SR3600; Sports-radar, Homosassa, FL) was used to measure the peak ball velocity and was placed in the line of the ball displacement. In order to perform the forehand and backhand test, balls were thrown by a ball machine (Lobster Elite V, Lobster Sports, Inc., North Hollywood, CA) at the mean of 73.3 ± 1.2 km·h^-1^ with no rotational effect and at a period of ten seconds per ball (6 shots·min^-1^). International Tennis Federation approved balls (Head ATP, Spain) were used and they were new in each testing session.

**Fig 2:**
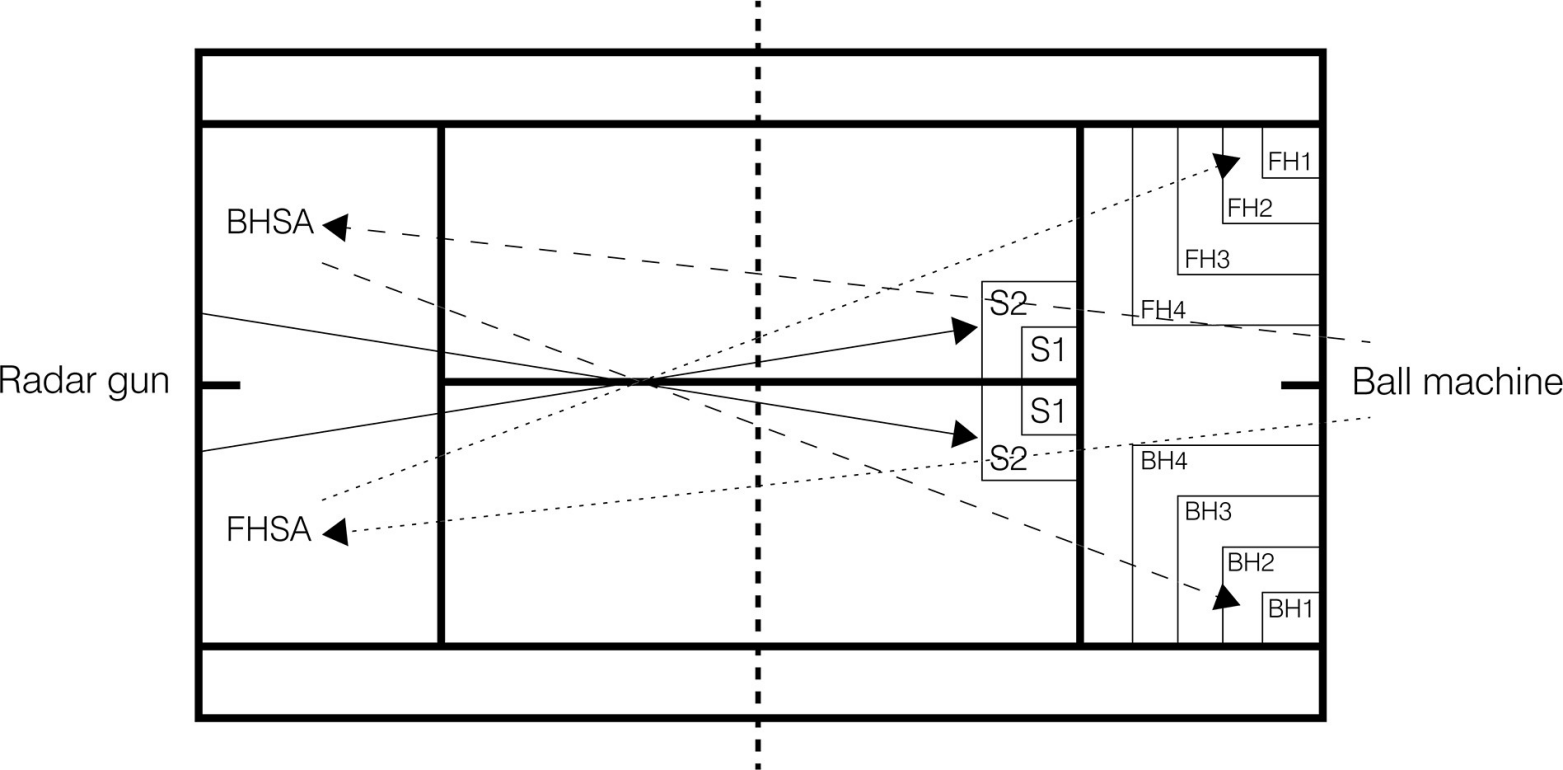
Stroke performance test [23]. S = serve; FH = forehand; BH = backhand. S1 and S2: Target area for the serve; FH1, FH2, FH3 and FH4: Target area for the forehand; BH1, BH2, BH3 and BH4: Target area for the backhand; FHSA: Forehand stroke impact area; BHSA: Backhand stroke impact area. Continuous line: Serve trajectory; dot line: Forehand trajectory; dash line: Backhand trajectory.

To evaluate stroke accuracy, Pialoux et al. [23] stroke performance design was followed (Fig 2):

Serve (S): S1 zone (0.5m*0.5m) 5 points; S2 zone (1m*1m) 3 points; rest of the serve box 1 point. Bounce ball out of serve box 0 points.

Baseline shots, forehand (FH) and backhand (BH): FH1 or BH1 zone (1m*1m) 5 points; FH2 or BH2 zone (2m*2m) 4 points; FH3 or BH3 zone (3m*3m) 3 points; FH4 or BH4 zone (4m*4m) 2 points; rest of the single court 1 point. Bounce ball out of single court 0 points.

Test reliability was good to excellent for stroke velocities (serve: intraclass correlation coefficient [ICC] = 0.967; forehand: ICC = 0.810; backhand: ICC = 0.904), limited for the groundstrokes accuracy (forehand: ICC = 0.468; backhand: ICC = 0.467) and poor for the serve accuracy (ICC = 0.275).

### Protocols

Both strength protocols are shown in Table 2. Maximal intended velocity training was fulfilled to induce greater strength gains [20]. The exercises in both methods used the main muscles involved in the strokes kinetic chain [1, 2]. The protocol for the MB was designed following the guidelines of Cardoso [1], Fernández-Fernández et al. [6] and Terraza-Rebollo et al. [12]. According to these guidelines, the subjects performed 3 sets of 6 repetitions of the exercises shown in Table 2 with a 2 kg medicine ball. Rest duration was 1 minute between sets and 3 minutes between rounds. MB total volume was 144 repetitions in 24 sets, having a session total tonnage of 288 kg (21 sets counting only one side of a one-arm overhead forward throw). In the case of the RT protocol, Cardoso [1], Faigenbaum et al. [24] and Terraza-Rebollo et al. [12] guidelines were followed. According to these guidelines, the subjects performed 3 non-consecutive sets of 6 repetitions with the 75% 1RM at maximal intended velocity for concentric contraction of the exercises shown in Table 2. Repetitions were prescribed in order to avoid failure. Rest duration was 2 minutes between sets and 3 minutes between rounds. Total volume was 90 repetitions in 15 sets (12 sets counting only one side of the one hand row), having a session total tonnage mean of 3450.6 ± 924.8 kg.

**Table 2:** Medicine ball throws (MB) and resistance training (RT) exercises.

MB							RT					
Exercise	Sets (no.)	Reps (no.)	Weight (kg)	Int vel	Rest set/round (min)	Exercise	Sets (no.)	Reps (no.)	1RM (%)	Int vel*	Rest set/round (min)
Forehand throw	3	6	2	Max	1 / 3	Bench press	3	6	75	Max	2 / 3
Backhand throw	3	6	2	Max	1 / 3	Half squat	3	6	75	Max	2 / 3
Chest throw	3	6	2	Max	1 / 3	One hand row**	3	6	75	Max	2 / 3
Two-arm overhead forward throw	3	6	2	Max	1 / 3	Dead lift	3	6	75	Max	2 / 3
Two-arm overhead backward throw	3	6	2	Max	1 / 3						
Two-arm overhead upward throw	3	6	2	Max	1 / 3						
One-arm overhead forward throw	3	6**	2	Max	1 / 3						

Reps = number of repetitions; Int vel = intended velocity; Rest set/round = rest between sets / rest between rounds

*concentric contraction

**each hand; 1RM = one repetition maximum; Max = maximal.

### Statistical analyses

Descriptive data were reported as mean ± standard deviation. The normality of the distributions and homogeneity of variances were assessed with the Shapiro-Wilk test. Intrasession reliability on the serve velocity and accuracy was determined using a two-way average measure of the ICC and refer to intra-subject variation between 6 measurements. The differences between velocity measured from the basal situation and at various recovery times (3 minutes [Post], 24 hours [Post24], 48 hours [Post48]) after performing RT and MB were evaluated using a repeated-measures analysis of variance (ANOVA), with Bonferroni-corrected post hoc analysis. Due to the fact that stroke accuracy data were not normally distributed, Friedman’s test was used to examine the differences at various times during recovery. The magnitude of the differences in mean was quantified as effect size (ES) and interpreted according to the criteria used by Cohen [25]: 0.20 to 0.49 = small; 0.50 to 0.79 = moderate; or >0.79 = large. Values lower than 0.20 were called as trivial since that no classification was attributed by Cohen [25] to these values. The level of significance was set at p < 0.05. Statistical analyses were performed using SPSS software (version 23.0; SPSS Inc, Chicago, IL).

## Results

The mean peak ball velocity and accuracy, effect size, mean change and percentage of changes of serve, forehand and backhand after the training protocols are shown in Table 3 for velocity and Table 4 for accuracy. There were no significant differences in ball velocity (MB serve: F_3,27_ = 1.076, *p* = 0.376, MB forehand: F_3,27_ = 1.451, *p* = 0.250; MB backhand: F_3,27_ = 1.633, *p* = 0.205; SC serve: F_3,27_ = 2.847, *p* = 0.056; SC forehand: F_3,27_ = 0.984, *p* = 0.415; SC backhand: F_3,27_ = 1.772, *p* = 0.176) (Table 3) and accuracy (MB serve: *p* = 0.633; MB forehand: *p* = 0.843; MB backhand: *p* = 0.530; SC serve: *p* = 0.300; SC forehand: *p* = 0.988; SC backhand: *p* = 0.651) (Table 4) following each time recovery (Post, Post24 and Post48), and for all strokes compared to the baseline in both training methods.

**Table 3:** Acute (Post) and delayed (Post24 and Post48) effects of medicine ball throws training (MB) and resistance training (RT) on ball velocity and corresponding percentage changes from baseline.

		Pre	Post	Difference			Post24	Difference			Post48	Difference		
		Velocity (Km/h)	Velocity (Km/h)	ES	Change (%)	Mean change (km/h)	Velocity (Km/h)	ES	Change (%)	Mean change (km/h)	Velocity (Km/h)	ES	Change (%)	Mean change (km/h)
**MB**	**Serve**	138.8 ± 15.7	139.4 ± 17.7	-0.20	0.8	1.1 ± 5.1	139.6 ± 15.9	-0.19	0.9	1.3 ± 2.3	140.8 ± 15.2	-0.61	1.8	2.5 ± 4.2
	**Forehand**	118.4 ± 9.5	118.2 ± 10.7	0.05	-0.2	0.2 ± 4.0	121.3 ± 11.5	-0.42	2.5	2.9 ± 7.0	117.8 ± 12.5	0.10	-0.5	-0.6 ± 6.0
	**Backhand**	113.2 ± 10.1	111.5 ± 11.8	0.38	-1.5	-1.7 ± 4.6	115.1 ± 8.5	-0.62	1.6	1.9 ± 3.0	111.8 ± 12.0	0.22	-1.2	-1.4 ± 6.3
**RT**	**Serve**	145.6 ± 21.4	141.6 ± 19.3	0.79	-2.7	-4.0 ± 5.0	145.7 ± 22.5	-0.02	0.1	0.1 ± 6.1	145.1 ± 23.4	0.14	-0.3	-0.5 ± 3.5
	**Forehand**	123.1 ± 14.1	121.1 ± 11.9	0.47	-1.6	-2.0 ± 4.2	119.5 ± 12.5	0.64	-2.9	-3.6 ± 5.7	121.3 ± 15.9	0.29	-1.5	-1.8 ± 6.3
	**Backhand**	110.6 ± 10.2	108.2 ± 8.7	0.56	-2.2	-2.4 ± 4.3	111.0 ± 9.6	-0.13	0.4	0.5 ± 3.5	108.6 ± 13.6	0.40	-1.8	-1.9 ± 4.8

Values are mean ± standard deviation. MB = medicine ball training; RT = resistance training; ES = effect size. Magnitudes of ESs were assessed using the following criteria: <0.20 = trivial, 0.20 to 0.49 = small, 0.5 to 0.79 = moderate, >0.79 = large.

**Table 4:** Acute (Post) and delayed (Post24 and Post48) effects of medicine ball throws training (MB) and resistance training (RT) on accuracy and corresponding percentage changes from baseline.

		Pre	Post	Difference			Post24	Difference			Post48	Difference		
		Velocity (points)	Velocity (points)	ES	Change (%)	Mean change (points)	Velocity (points)	ES	Change (%)	Mean change (points)	Velocity (points)	ES	Change (%)	Mean change (points)
**MB**	**Serve**	0.68 ± 0.31	0.56 ± 0.24	0.26	-14.8	-0.10 ± 0.36	0.61 ± 0.23	0.18	-9.8	-0.07 ± 0.45	0.78 ± 0.23	-0.30	16.1	0.11 ± 0.37
	**Forehand**	1.52 ± 0.56	1.55 ± 0.49	-0.04	2.0	0.03 ± 0.73	1.56 ± 0.40	-0.05	2.6	0.04 ± 0.81	1.62 ± 0.59	-0.16	6.6	0.10 ± 0.61
	**Backhand**	1.65 ± 0.43	1.69 ± 0.40	-0.06	2.4	0.04 ± 0.63	1.91 ± 0.56	-0.75	15.8	0.26 ± 0.35	1.85 ± 0.32	-0.32	12.1	0.20 ± 0.62
**RT**	**Serve**	0.70 ± 0.36	0.58 ± 0.36	0.51	-17.9	-0.13 ± 0.28	0.78 ± 0.53	-0.14	10.7	0.08 ± 0.49	0.51 ± 0.31	0.66	-27.4	-0.19 ± 0.29
	**Forehand**	1.65 ± 0.54	1.57 ± 0.49	0.18	-4.9	-0.08 ± 0.45	1.74 ± 0.63	-0.25	5.5	0.09 ± 0.37	1.43 ± 0.42	0.29	-13.3	-0.22 ± 0.75
	**Backhand**	1.47 ± 0.62	1.51 ± 0.50	-0.07	2.7	0.04 ± 0.56	1.73 ± 0.45	-0.52	17.7	0.26 ± 0.50	1.53 ± 0.60	-0.11	4.1	0.06 ± 0.54

Values are mean ± standard deviation. MB = medicine ball training; RT = resistance training; ES = effect size. Magnitudes of ESs were assessed using the following criteria: <0.20 = trivial, 0.20 to 0.49 = small, 0.5 to 0.79 = moderate, >0.79 = large.

## Discussion

The aim of this study was to investigate the acute (3 min) and delayed (24 and 48 h) effects of explosive training (2 kg MB) and resistance training (75% 1RM) in ball velocity and accuracy in young competition tennis players. The main results reported that MB and RT do not have any acute and delayed effect in serve, forehand and backhand ball velocity and accuracy. On the contrary, a common belief among tennis players and coaches is that strength training could impair ball velocity and accuracy in their acute and delayed effects.

Although this investigation did not find any negative effect in the stroke ball velocity and accuracy in young competition tennis players, it has been observed an acute performance decrease performing an explosive strength training (plyometric training or resistance training at 40% 1RM) [17, 18, 26] and maximal strength training (isometrics contraction at maximal voluntary contraction or dynamics contractions at 80 to 100% 1RM) [18–20, 27]. Thomas et al. [19] observed maximum voluntary contraction loss performing of 10 x 5 repetitions at 80% 1 RM of back squat (similar to our protocol), which could be recovered at 72 hours after training. Moreover, a decrease was observed in power, repeated-sprint ability, and shot accuracy in semi-professional male basketball players performing heavy RT, but this protocol performed 6 repetitions to failure [28]. However, similar to our protocol, it has not been observed changes in vertical jump, anaerobic power, or shot accuracy performing RT (5–12 repetitions at 60–80% 1RM) in collegiate women basketball players after 6 hours of recovery time [29]. This disparity of results may be explained by the different training variables performed in the investigations such as volume, intensity, and rest duration. In our investigation the subjects did not perform the repetitions to failure in order to properly perform strength training for young athletes [24], and the rest interval between sets was enough to avoid the excessive neuromuscular fatigue and to maintain the number of repetitions per set [24, 30]. Although repetition to failure is commonly used in strength training [31], it is not a critical aspect to enhance muscle hypertrophy or strength [32], and it could cause a phenotype muscle change to slower fibers [31, 33] and an increase of blood ammonia level [32, 34]. Besides, repetition to failure could increase the time needed for the recovery of neuromuscular function and metabolic and hormonal homeostasis [35].

Even though this investigation did not find a performance stroke decrease, neuromuscular fatigue could happen due to the fact that the central nervous system uses different neuromuscular strategies to overcome the fatigue in order to maintain the performance [36]. Similarly, during a tennis match, it has been shown technical impairments with no effect in serve velocity and accuracy [37] and both remaining stable during five-set professional matches on grass surface [38]. Moreover, during a three-day tennis tournament, a decrease in lower extremities of RFD and maximal voluntary contraction was found after the first day, but the serve velocity remained stable until before the third match, and no effects were found in countermovement jump [39]. In addition, it has been observed that expert players could have less fatigue’s detrimental effects on accuracy in tennis [40] and in table tennis [41] suggesting the theory of strategies to compensate for the fatigue effects [36, 41]. Therefore, in spite of not measuring the RFD, muscle activity or the maximal voluntary contraction, it could be hypothesized that neuromuscular fatigue with no effect in ball velocity could have happened in this investigation, involving strategies to compensate the fatigue effects.

It should be taken into consideration that after performing RT protocol in Post, a velocity decrease with moderate effect size was observed when performing serve (-2.7%) and when performing backhand (-2.2%). In addition, with the same protocol and recovery time, a decrease in accuracy with moderate effect size was found when performing serve (-17.9%). These results could suggest that RT causes a higher impairment in stroke performance and, as a consequence, it is important to be more careful when applying RT method, especially close to the competition.

The poor accuracy reliability in serve could be due to the greater technical difficulty of serve than groundstrokes. Also, it was probably due to the subjects were asked to hit the ball at maximum velocity, causing more variability in the accuracy and consequently decreasing the reliability. This fact has been observed in tennis [16, 42] and dart throwing [43], though there is some controversy because it has also been observed a positive correlation between velocity and accuracy in tennis [44].

The main limitations of the investigation were: that we investigated both genders together (it has been observed different acute effects in strength training by gender [18]); that there was a low reliability in the accuracy test; that the way to obtain 1RM was estimated; and that the volume of the strength training protocols were not equalized. Concerning the last point, volume training protocols were not equalized because common authors guidelines were followed to design them [1, 6, 12, 24]. Further research should focus on investigating the effects on neuromuscular fatigue and it would be interesting to study the effects of new strength training devices such as isoinertial training.

Regarding practical applications, it could be suggested that these two strength training methods (RT at 75% 1RM and 2 kg MB) might be useful to train the maximum and explosive strength [1, 12] without ball velocity and accuracy decrease and could be used before a technical-tactical session on-court or off-season, and in-season. It is advisable to avoid the repetitions to failure and perform the movement at maximal intended velocity. Coaches should take into account that these results are exclusively for these training methods and 1RM was estimated.

## Conclusion

Overall, the findings of the present research indicate that MB and RT, avoiding repetition to failure and at maximal intended execution, have no acute and delayed detrimental effects on stroke tennis performance. Therefore, it could be suggested that these two strength training methods using these protocols might be useful to train the maximum and explosive strength without ball velocity and accuracy decrease and could be used before a technical-tactical session on-court or off-season, and in-season.

## Supporting information

S1 Data (XLSX)
